# Early life diet conditions the molecular response to post-weaning protein restriction in the mouse

**DOI:** 10.1186/s12915-018-0516-5

**Published:** 2018-05-02

**Authors:** Amy F. Danson, Sarah J. Marzi, Robert Lowe, Michelle L. Holland, Vardhman K. Rakyan

**Affiliations:** 1 The Blizard Institute, Queen Mary University of London, 4 Newark Street, London, E1 2AT UK; 2 Department of Medical and Molecular Genetics, King’s College London, Guys Hospital, London, SE1 9RT UK

**Keywords:** Nutrition, protein restriction, ribosomal DNA, DNA methylation, small RNA, mismatch

## Abstract

**Background:**

Environmental influences fluctuate throughout the life course of an organism. It is therefore important to understand how the timing of exposure impacts molecular responses. Herein, we examine the responses of two key molecular markers of dietary stress, namely variant-specific methylation at ribosomal DNA (rDNA) and small RNA distribution, including tRNA fragments, in a mouse model of protein restriction (PR) with exposure at pre- and/or post-weaning.

**Results:**

We first confirm that pre-weaning PR exposure modulates the methylation state of rDNA in a genotype-dependent manner, whereas post-weaning PR exposure has no such effect. Conversely, post-weaning PR induces a shift in small RNA distribution, but there is no effect in the pre-weaning PR model. Intriguingly, mice exposed to PR throughout their lives show neither of these two dietary stress markers, similar to controls.

**Conclusions:**

The results show that the timing of the insult affects the nature of the molecular response but also, critically, that ‘matching’ diet exposure either side of weaning eliminates the stress response at the level of rDNA methylation and small RNA in sperm.

**Electronic supplementary material:**

The online version of this article (10.1186/s12915-018-0516-5) contains supplementary material, which is available to authorized users.

## Background

Various environmental factors, such as levels of physical activity or poor diet, can potentially influence health and disease states in mammals. As environmental stressors can operate at any point during the life course [1], it is necessary to understand how the timing of exposure to these factors influences molecular responses. Within this context, understanding the dynamics of molecular markers of the stress response would greatly enhance the ability to monitor the impact of environmental stressors in mammals and, ultimately, to gain mechanistic insight into the stress pathways involved.

**Fig. 1:**
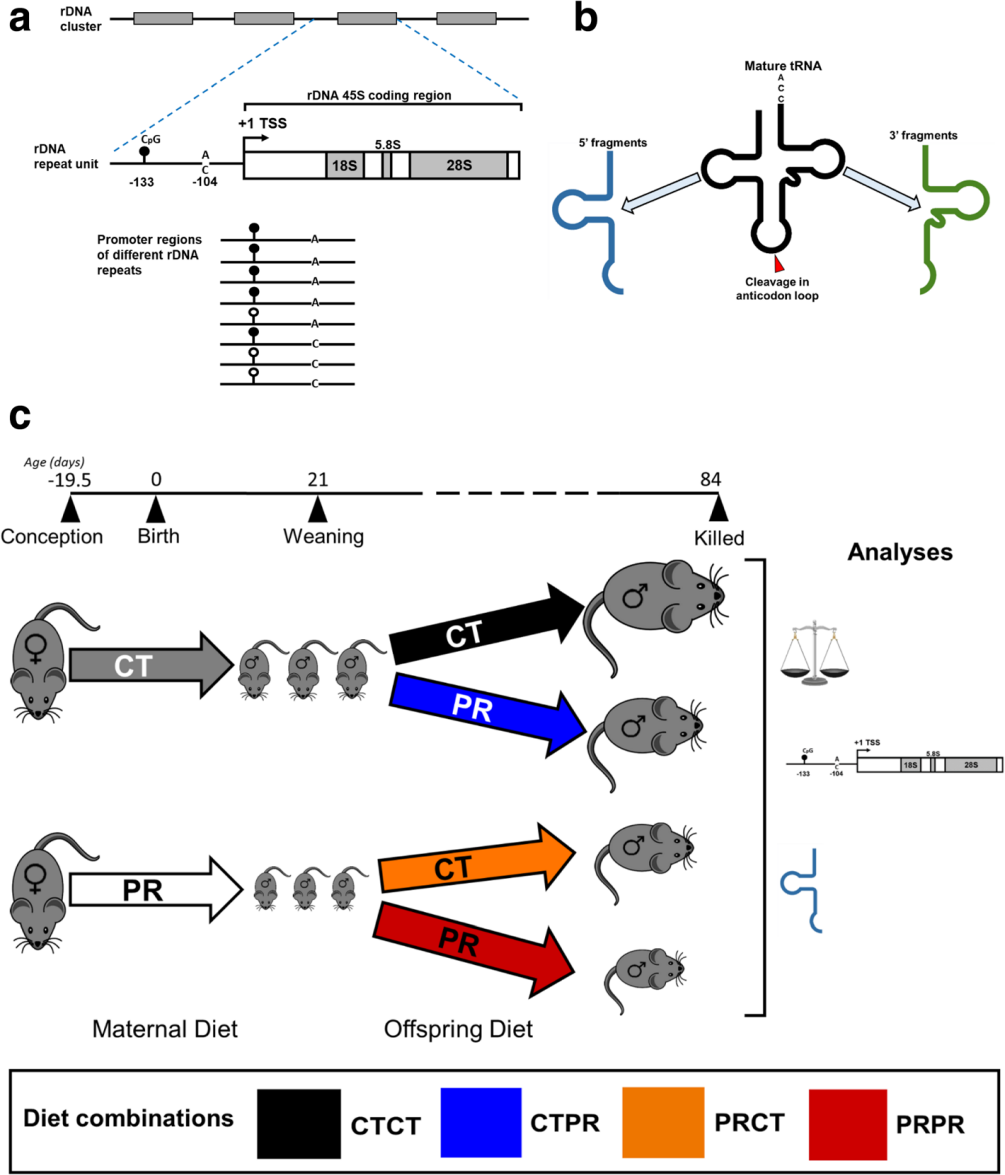
A C57BL/6 J protein restriction mouse model that combines two previously studied models with contradictory molecular consequences. **a** BisPCR-seq was used to simultaneously analyse methylation at CpG −133 (methylation = black circles) and genetic variation at the A/C SNP at position −104 bp in the promoter region of the 45S rDNA tandem repeats. **b** Small RNA-seq was used to analyse tRNA fragments derived from cleaved mature tRNAs. **c** Breeding and experimental scheme. Pregnant female C57BL/6 J mice were put on either a control (CT, 20% protein) or protein restricted (PR, 8% protein) diet from conception to the end of lactation. Male offspring in each litter were put on either a CT or PR diet from weaning to give four diet combinations – CTCT (black), CTPR (blue), PRCT (orange), PRPR (red). As in previously studied models, phenotype, rDNA methylation and tRNA fragment analyses were performed on sperm and other tissues taken at 84 days of age

Recently, we reported that protein restriction (PR) in mice from conception until weaning induced a linear correlation between growth in early life and DNA methylation within the ribosomal DNA (rDNA) promoter [2]. rDNA codes for the ribosomal RNA that contributes to the structure of the ribosome and exists in the genome in tandem repeats at multiple loci (Fig. 1a) [3]. We found a significant negative relationship between mice weaning weight and methylation status of a specific functional CpG, 133 bp upstream of the transcriptional start site of the rDNA locus (CpG −133), the methylation of which is associated with suppression of transcription of that particular rDNA gene copy [2, 4]. However, this relationship was only observed in mice exposed to in utero PR, and not in controls [2]. This epigenetic response remained into adulthood, even after the PR mice were put on a control diet after weaning. Crucially, this response only occurred at a subset of the rDNA copies within the mouse genome, specifically those containing an ‘A’ base at position 104 bp upstream of the TSS (Fig. 1a). rDNA copies with ‘C’ at this position did not show environment-induced methylation dynamics at CpG −133. Furthermore, the epigenetic state correlated with both transcriptional and phenotypic outcomes, and hypermethylation of rDNA was also identified after exposure to both high-fat and obesogenic diets from conception to weaning [2]. Collectively, these results identified a mammalian example of epigenetic dynamics induced by an interaction between the genotype and the early life environment. Mouse rDNA hypermethylation in response to in utero PR exposure was also independently demonstrated by Denisenko et al. [5].

On the other hand, Shea et al. [6] reported a mouse model in which they exposed mice to a PR diet from weaning onwards, and although they observed substantial genetic and epigenetic heterogeneity at rDNA, there were no observable diet-specific effects. Both our group [2] and Shea et al. [6] examined inbred C57BL/6 J mice and used similar PR exposures. Although part of the reason for the discrepant observations between these two studies could be that Shea et al. [6] did not discriminate between the A or C genetic variants, another potential explanation lies in the differences in the timing of PR exposure. Interestingly, using a similar post-weaning PR exposure mouse model (albeit in a different mouse strain), the authors subsequently reported a marked increase in transfer RNA (tRNA) fragments that result from the cleavage of mature tRNAs at specific sites [7] (Fig. 1b). Separately, Chen et al. [8] reported tRNA fragments to be increased in the sperm of male mice after being fed a high-fat diet post-weaning.

Although valuable insights have been gained by previous genomic and epigenomic analyses performed in the context of dietary models in rodents [9–13], what is noteworthy about the findings related to rDNA and tRNA fragments is that they have been reported by independent groups in different dietary exposure models [2, 5, 7, 8, 14]. Indeed, both rDNA and tRNA fragments have been implicated in cellular stress response mechanisms that are conserved amongst species [15–20]. Given the potential of rDNA and tRNA fragments to be robust ‘molecular barometers’ of dietary stress in different experimental models, including rodents, we set out to address three key inter-related questions raised by the recent studies described above. Firstly, is the difference in rDNA responses between our previous model [2] and that of Shea et al. [6] due to the timing of the PR exposure? Secondly, are tRNA fragments upregulated when the environmental challenge is experienced during early life? And finally, what happens when the animal is exposed to poor nutrition throughout the life-course? Answers to these questions would, in a more general sense, provide an enhanced understanding of how the timing of environmental exposures impacts the dynamic molecular responses of a mammalian genome.

## Results

Pregnant inbred C57BL/6 J mice were fed either a control (CT, 20% protein, 11 litters) diet or a PR (8% protein, 10 litters) diet throughout pregnancy and lactation (Additional file 1: Table S1). All litters were derived from independent females, i.e., no females were used to generate more than one litter. At weaning (3 weeks), the male offspring from each litter were assigned to either the CT or PR diet until they were killed at 11–13 weeks of age (Fig. 1c). Four diet combinations were therefore studied, namely (1) CT throughout life (CTCT, *n*_*Litters*_ = 10), (2) CT pre-weaning followed by PR post-weaning (CTPR, as in Shea et al. [6], *n*_*Litters*_ = 11), (3) PR pre-weaning followed by CT post-weaning (PRCT, as in Holland et al. [2]*,* 2016, *n*_*Litters*_ = 9) and (4) PR throughout life (PRPR, *n*_*Litters*_ = 10).

**Fig. 2:**
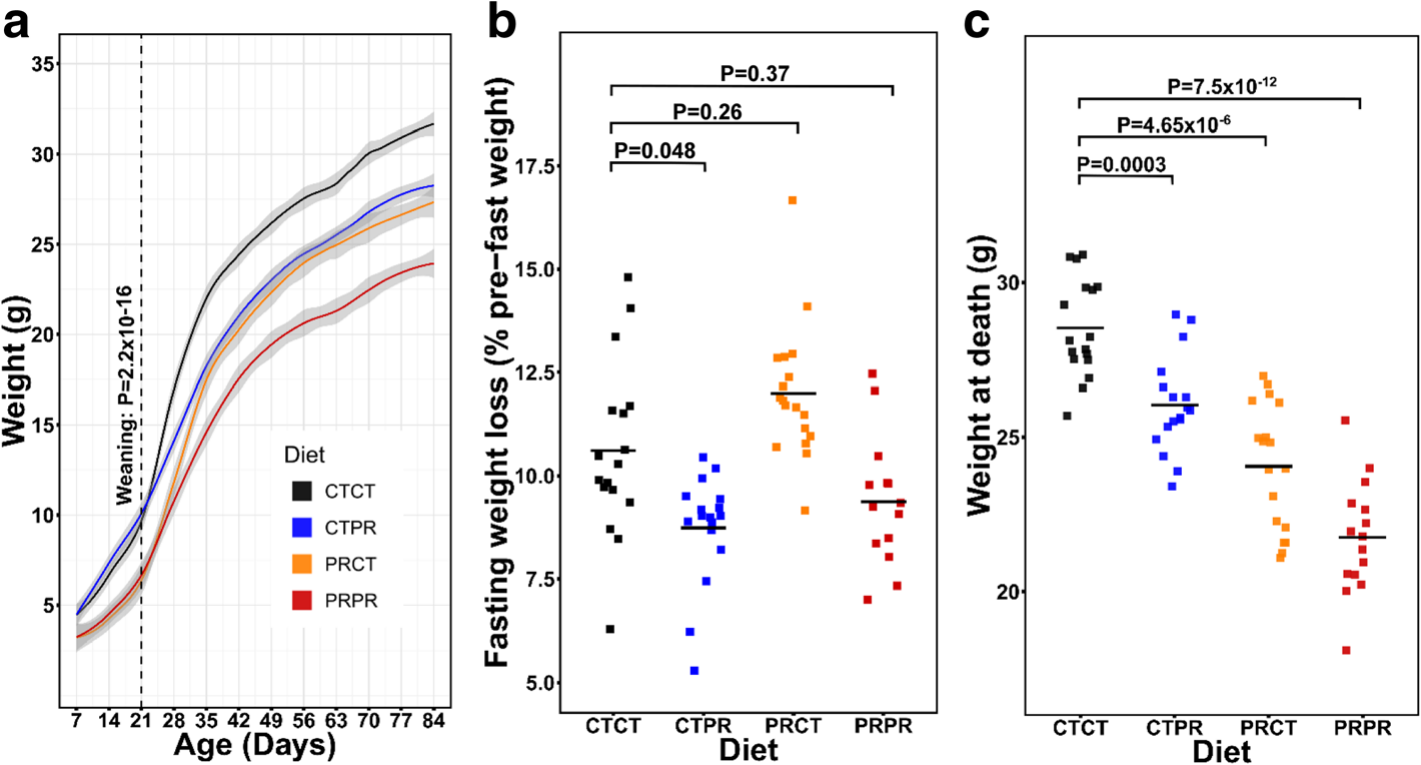
A C57BL/6 J protein restriction mouse model that replicates phenotypes seen previously. **a** Weight progression of male offspring between 7 and 84 days of age. Grey shading = 95% confidence interval. Dotted line = time of weaning. Weaning weights of male offspring exposed to PR diet (*n* = 33, *n*_*Litters*_ = 10) were 34% lower than those exposed to CT diet (*n* = 34, *n*_*Litters*_ = 11). *P* = 2 × 10^− 16^, Welch’s *t* test using litter means. **b** Fasting weight loss during 16 h fast as a proportion of pre-fast weight was lower in CTPR (mean = 8.87%, *n* = 17, *n*_*Litters*_ = 11) than CTCT (mean = 10.6%, *n* = 17, *n*_*Litters*_ = 10) (*P* = 0.048) and the same in PRCT (mean = 11.99%, *n* = 18, *n*_*Litters*_ = 9) and PRPR (mean = 9.37%, *n* = 15, *n*_*Litters*_ = 10) as CTCT (*P* = 0.26 and *P* = 0.37). A linear model was run for each phenotype against diet and sibling relatedness was accounted for using robust standard errors. *P* values were adjusted for *n* = 3 tests using Bonferroni’s method. **c** Weight at death was distinct between the four diet groups. CTPR mice were significantly lighter (mean = 26.05 g) than CTCT mice (mean = 28.53 g) at death (*P* = 0.0003). PRCT mice were lighter than CTCT mice (mean = 24.06 g, *P* = 4.65 × 10^− 6^) and PRPR mice were the lightest (mean = 21.76 g, *P* = 7.5 × 10^− 12^). A linear model was run for each phenotype against diet and sibling relatedness was accounted for using robust standard errors. *P* values were adjusted for *n* = 3 tests using Bonferroni’s method

We previously demonstrated that body weight at weaning was significantly lower in offspring of mothers fed a PR diet [2]. Our data in the present study replicates this finding, as offspring exposed to pre-weaning PR were, on average, 34% lighter than the offspring of CT-fed mothers (Fig. 2a dotted line, *P* = 2.2 × 10^− 16^). After weaning, the growth rate of mice was determined by a post-weaning diet (Fig. 2a, Additional file 1: Figure S1a). Despite this, the absolute weight of the PRCT group did not catch up to that of the CTCT group, indicating that the post-weaning diet could not compensate for the growth retardation induced by the pre-weaning diet.

Mice were killed at 11–13 weeks of age, after overnight fasting. The death weights of the four diet groups were significantly different from each other (Fig. 2c; CTPR *P* = 0.0003, PRCT *P* = 4.65 × 10^− 6^, PRPR *P* = 7.5 × 10^− 12^) and this was because CTPR mice lost less weight than CTCT mice (Fig. 2b; *P* = 0.048), whilst weight loss in PRCT and PRPR mice was similar to the controls (*P* = 0.26 and *P* = 0.37, respectively). Interestingly, despite their differences in size, relative organ and fat deposit weights were the same between the CTCT and the PRPR groups (Additional file 1: Figures S2 and S3), except for the relative kidney weight, which was lower in the PRPR group (Additional file 1: Figure S2e).

Overall, the PR model reported here replicates the phenotypes seen previously by our group [2] and others in the PRCT [2, 21] and CTPR branches [22, 23]. We have extended the model to include the PRPR group, which, despite being the lightest in body mass, appears to differ little from the CTCT group in terms of phenotype, at least as measured between 11 and 13 weeks.

To study the molecular responses to differences in timing of PR exposure, we focussed on mature sperm, as this was the common tissue between the previous models from our group [2] and the model from Sharma et al. [7], thus permitting direct comparison. High sperm purity was consistently obtained (Additional file 1: Figure S4). Extracted DNA was sequenced by multiplex bisulfite PCR sequencing (‘BisPCR-Seq’), as in Holland et al. [2]. This method allowed quantification of the A/C genetic variant frequency at position −104 bp in the promoter region of rDNA and of the frequency of methylation at the functional CpG site at −133 bp.

**Fig. 3:**
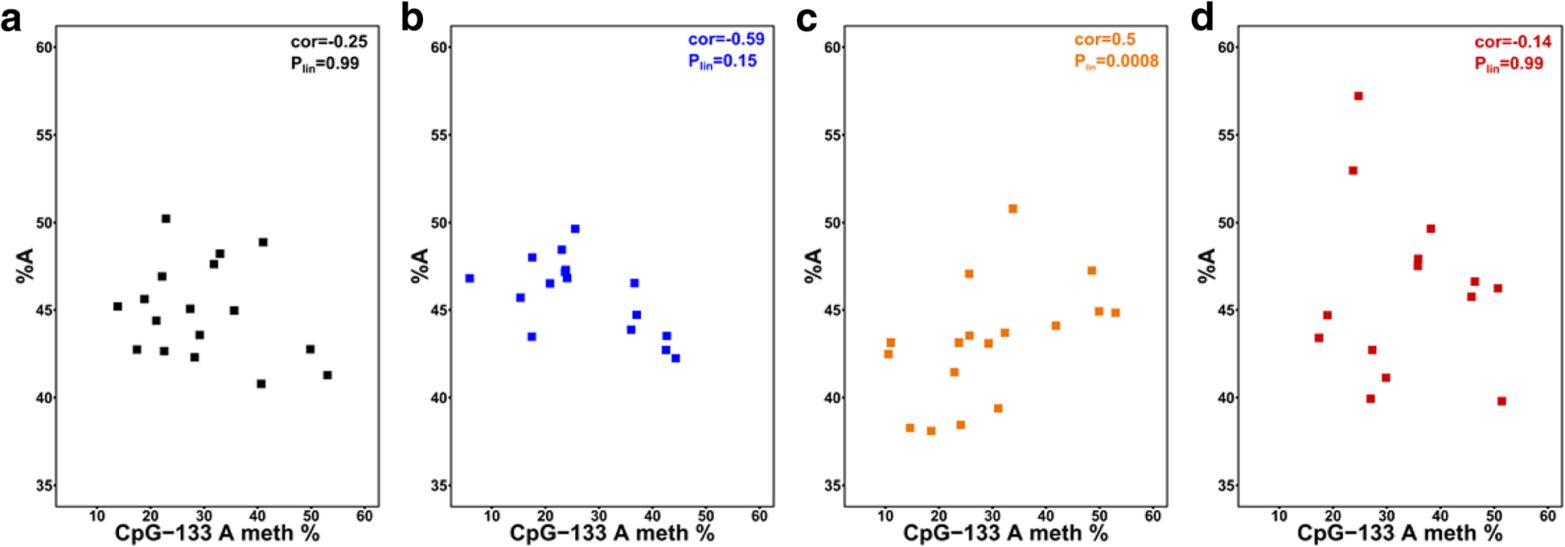
Molecular changes to variant-specific rDNA methylation response to protein restriction are restricted to pre-weaning exposure. **a** In sperm, percentage of rDNA copies with an A at position −104 bp (%A) does not correlate with percentage of those A copies with methylation at CpG −133 (CpG –133 A meth %) in CTCT (black, *n* = 17, *n*_*Litters*_ = 10, cor = −0.25, adjusted *P*_*lin*_ = 0.99). **b** %A in CTPR (blue, *n* = 17, *n*_*Litters*_ = 11, cor = −0.59, adjusted *P*_*lin*_ = 0.15). **c** %A correlates positively with CpG –133 A meth % in PRCT (orange, *n* = 16, *n*_*Litters*_ = 9, cor = 0.5, adjusted *P*_*lin*_ = 0.0008). **d** There was no correlation between %A and CpG –133 A meth % in PRPR (red, *n* = 14, *n*_*Litters*_ = 10, cor = −0.14, adjusted *P*_*lin*_ = 0.99). Pearson’s product moment correlation coefficients are given and a linear model was run to assess the relationship in group with sibling relatedness being accounted for using robust standard errors. *P* values are adjusted for *n* = 4 tests using Bonferroni’s method

In our previous work, only the CTCT and PRCT groups were examined. The key finding was that maternal PR induced a correlation between the relative number of rDNA copies with an ‘A’ at position −104 bp in an individual (%A) and the frequency of methylation of A variants at CpG −133 (CpG –133 A meth%, abbreviated to A_meth%_). The present study replicates these findings, with a positive correlation between %A and A_meth%_ in the PRCT group (Fig. 3c; PRCT orange, cor = 0.50; linear model with robust standard errors, *P*_*lin*_ = 0.0008) but not in the CTCT group (Fig. 3a; CTCT black, cor = −0.25; linear model with robust standard errors, *P*_*lin*_ = 0.99). We used all mice in this analysis (rather than litter averages) as well as a linear model with robust standard errors to correct for relatedness among littermates (see Methods). Expanding upon our previous work, we examined this relationship in the other dietary regimes in the present study. We observed no correlation between %A and A_meth%_ in the CTPR group (Fig. 3b; CTPR blue, cor = −0.59; linear model with robust standard errors, *P*_*lin*_ = 0.15), suggesting that exposure to PR pre-weaning is necessary for the induction of this particular DNA methylation response at rDNA. Surprisingly, there was no relationship between %A and A_meth%_ in the PRPR group (Fig. 3d; PRPR red, cor = −0.14; linear model with robust standard errors, *P*_*lin*_ = 0.99), suggesting that post-weaning PR may reverse the effect of pre-weaning PR. We conclude from these findings that rDNA epigenetic responses to malnutrition may also be modulated by a post-weaning diet.

**Fig. 4:**
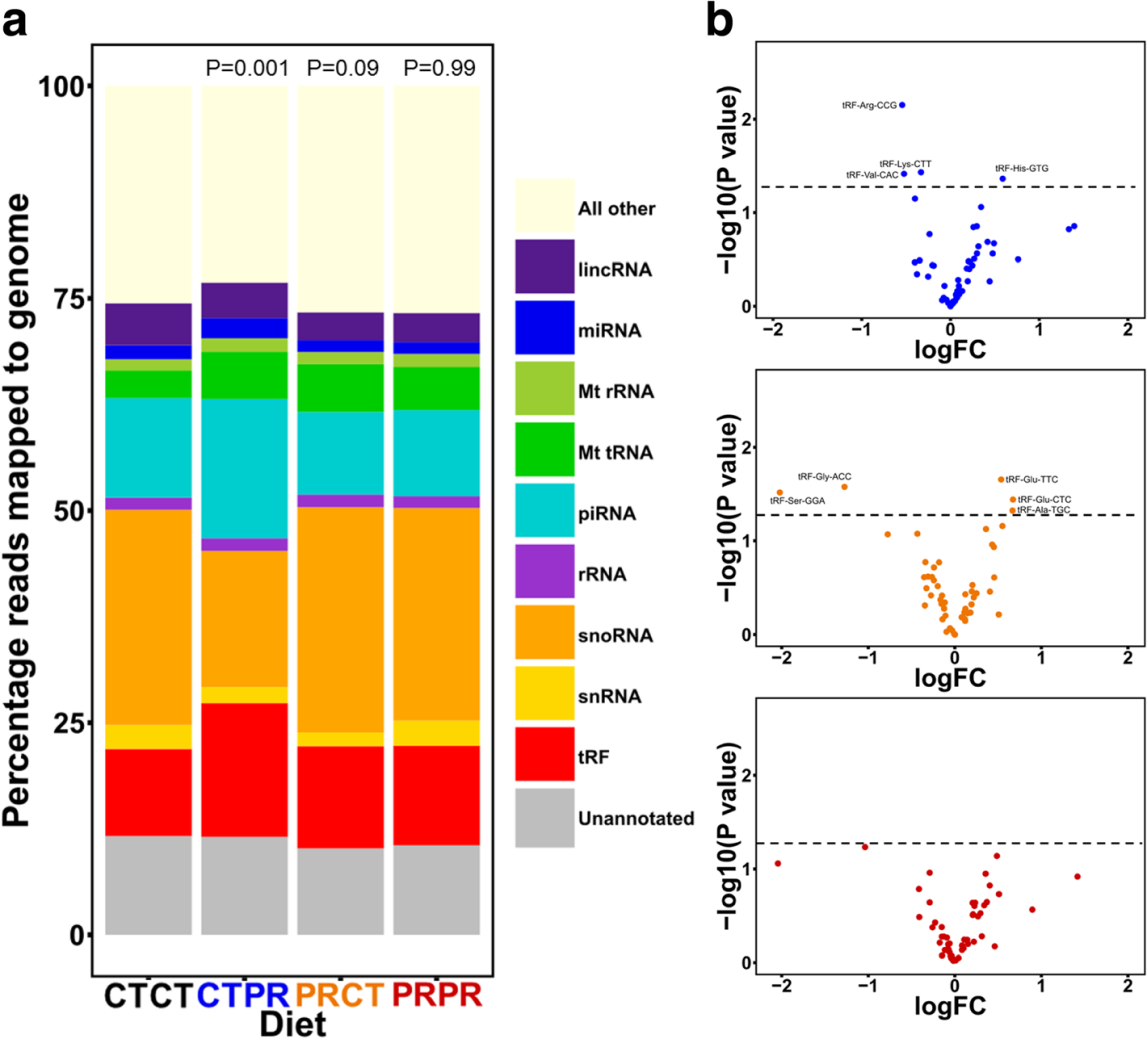
Post-weaning protein restriction leads to an altered small RNA profile in sperm but only when there has been no previous PR exposure. **a** The percentage of mappable reads falling into each class of small RNA, including transfer RNA (tRNA) fragments (tRF), small nuclear RNA (snRNA), small nucleolar RNA, ribosomal RNA (rRNA), Piwi-interacting RNA (piRNA), mitochondrial tRNA (Mt tRNA), mitochondrial ribosomal RNA (Mt rRNA), microRNA and long intergenic non-coding RNA (lincRNA). The percentage of unannotated reads are marked in grey and all other reads (processed transcripts and mRNA) are marked in beige. Fisher’s exact test for count data was used to assess the differences in distribution across the classes between the CTCT group and the CTPR group (adjusted *P* = 0.001), PRCT group (adjusted *P* = 0.09), and PRPR group (adjusted *P* = 0.99). *P* values were adjusted for *n* = 3 tests using Bonferroni’s method. **b** Differential expression analysis showed no genome-wide significant changes in abundances of tRNA-derived fragments between the CTCT group and any of the other groups (CTPR = blue, PRCT = orange, PRPR = red). The dotted line in each plot represents the nominal significance level (*P* = 0.05)

Having established that the rDNA variant-specific methylation changes in response to protein restriction occur during early life only, we next sought to analyse tRNA fragment and small RNA profiles in the four different groups. Small RNA was extracted from the same sperm samples and RNA-seq was performed. Reads were aligned to the whole genome and then to databases for tRNA, Piwi-interacting RNA (piRNA), microRNA and other small RNA categories. Figure 4a shows the percentage of mappable reads falling into each small RNA category in the four groups. The distribution of reads across the different small RNA classes was significantly different in the CTPR group from the distribution in the CTCT group (Fisher’s exact test, *P* = 0.001) but not different in the PRCT (*P* = 0.09) or PRPR groups (*P* = 0.99). In particular, the differences in the CTPR group appeared to be driven by differences in reads aligning to cellular tRNA, mitochondrial tRNA, piRNA and small nucleolar RNA. Length distribution analyses indicated that the majority of reads aligning to tRNA were between 28 and 34 nucleotides in length and were therefore considered to be tRNA fragments (Additional file 1: Figure S5). Differential expression analysis of reads that mapped to tRNA showed that there were more differences in tRNA fragments between the controls and the CTPR and PRCT groups (Fig. 4b, nominally significantly different tRNA fragments lie above the dotted line and are labelled) than the PRPR group but none of these changes reached genome-wide significance. Overall, although statistical analysis of individual small RNA groups, such as tRNA fragments, did not show the differences between groups seen by Sharma et al. [7], it is clear that there is a change in small RNA composition in response to post-weaning PR, which is not seen in response to pre-weaning PR. Interestingly, these changes are also not seen in the PR throughout life group (PRPR), suggesting that small RNA changes are an acute response and that early-life PR may be ‘protective’ of further changes on continued exposure.

## Discussion

Genetic–epigenetic interactions at rDNA and an increase in tRNA fragments have been shown to represent molecular markers of dietary stress (such as PR) in different mouse models [2, 5, 7, 24]. The model used herein includes a re-examination of previously published pre- or post-weaning only PR exposures (PRCT and CTPR, respectively), and reveals the following key observations. Firstly, we confirm that rDNA variant-specific methylation effects are induced when PR exposure occurs pre-weaning only but not when it occurs post-weaning only and, secondly, that the CTPR group was the only one of the three different exposure groups that displayed a significant redistribution of the relative proportion of mapped tRNA fragments, small nucleolar RNAs and piRNAs. We were unable to reproduce the relative increase in specific tRNA fragments in the CTPR group as reported by Sharma et al. [7], but this may simply reflect natural experimental variation between our iteration of the model versus theirs, or subtle but relevant differences between our C57BL/6 J mice and the FVB/NJ mice used therein. However, the data do support the broader conclusion that post-weaning dietary stress induces perturbation of small RNAs (at least in sperm), whereas this is not observed in the pre-weaning PR exposure. Collectively, these results address the first two aims of our study, and further underline the robustness of rDNA and tRNA as ‘molecular barometers’ of dietary stress in mouse models.

**Fig. 5:**
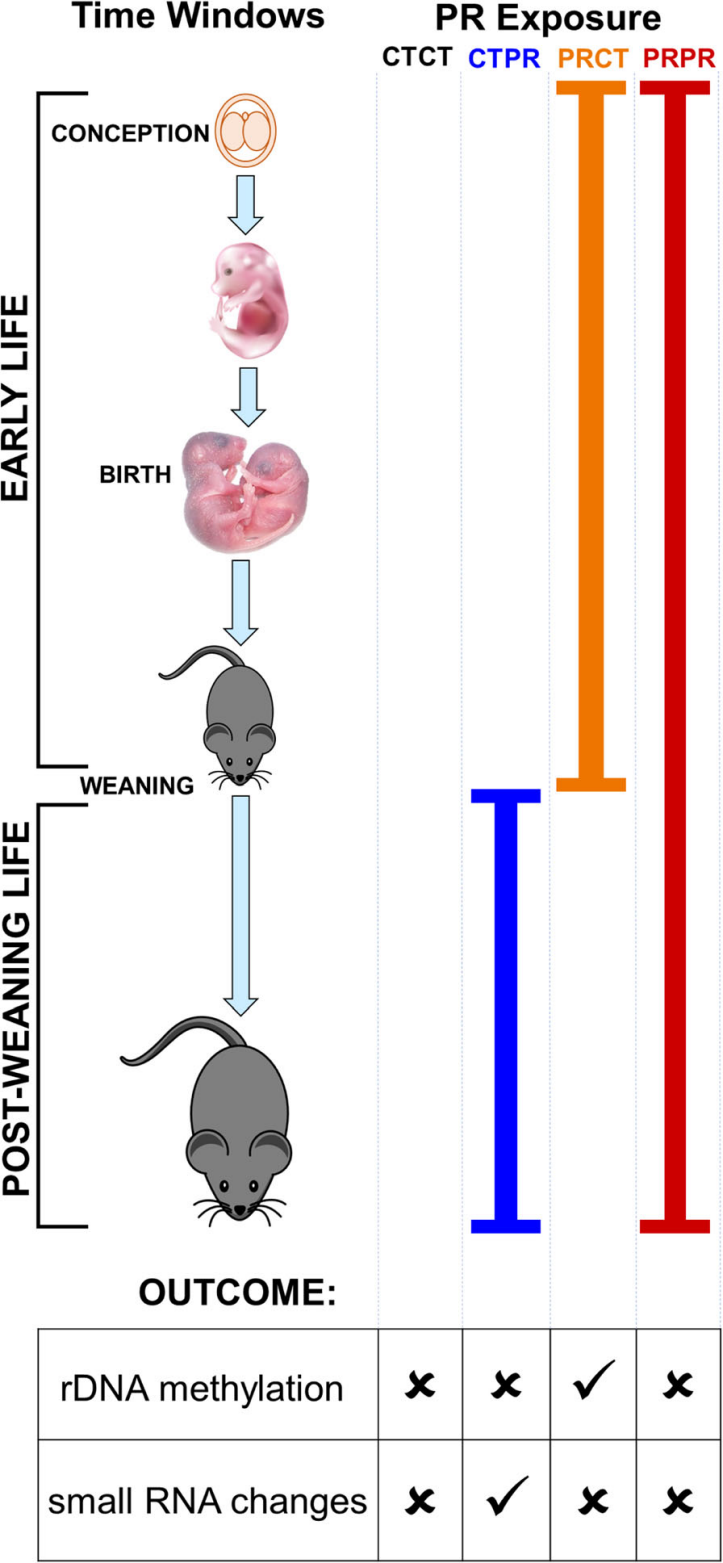
Early life diet conditions the response of rDNA and tRNA to post-weaning diet in a pre- and post-weaning protein restriction mouse model

The more remarkable finding relates to the molecular responses to persistent poor nutrition throughout life (PRPR group). Across both the rDNA and small RNA responses, the PRPR group is indistinguishable from the CTCT group (Fig. 5). Variant-specific rDNA methylation effects are absent in the PRPR group, all of whom are siblings of the mice in the PRCT group in which we see the distinctive relationship between %A and A_meth%_. This either suggests that the rDNA methylation response is established at weaning and later ‘reversed’ in the PRPR group during post-weaning life, or that the response is established in adulthood in the PRCT group only. In either case, the variant-specific rDNA methylation effect is a reflection of the diet combinations across the whole life-course of the animal; that is to say, it is the result of adulthood exposure to a control diet given that the animal was exposed to PR during early life (PRCT). Furthermore, changes in the relative proportions of small RNAs are seen in response to post-weaning PR only (CTPR), but are largely absent from the PRPR group (Fig. 5). Pre-weaning PR may therefore be ‘protective’ against a small RNA stress response on exposure to further PR during adulthood. Taken together, we conclude that the rDNA and small RNA responses observed herein are a consequence of the combined effects of control and PR diets during particular time windows across the entire life-course. Our results are reminiscent of observations made in human studies, in which mismatch between early life and adulthood diets lead to adverse metabolic outcomes in later life [25, 26]. It will therefore be interesting to test whether such perturbations at rDNA and small RNAs are also found in these affected human populations.

There are some limitations to our work that can be addressed in future studies. First, it is well established that the birth-to-weaning time window is another critical period in the life-course of the mouse [27], with growth and development of many neuroendocrine systems occurring in this period in mice as would occur in utero in humans [28]. No single model can capture the nuances of mismatch between every critical developmental stage so the birth-to-weaning period will be an important model to address in the future, and this may also shed light on why the relationship between %A and A_meth%_ is not observed in PRPR mice. Second, it will be important to analyse the role that rDNA and small RNA perturbations play in the development of disease phenotypes using dedicated models in which one would specifically modulate, for example, specific small RNAs in vivo or alter the methylation of specific rDNA copies. These experiments would also need to be performed in tissues potentially more relevant to the phenotype under consideration, for example, in liver or adipose tissues, to investigate the potential downstream metabolic outcomes. We focused on sperm in this study allowing us to directly compare our previous model [2] and that from Sharma et al*.* [7], and because sperm can be isolated to very high degrees of purity, thus reducing the differential cell composition biases that can potentially arise when using more complex tissues (although it should be noted that we also observed the %A vs. A_meth%_ relationship in livers of PRCT mice [2]). Indeed, the mechanisms by which rDNA and small RNAs act as stress responses may be interconnected – it was recently found that certain tRNA fragments can modulate the expression of ribosomal proteins and therefore ribosome biogenesis [29]. In this case, we suggest that different molecular mechanisms could operate to bring about the same outcome (changes to ribosome biogenesis) when the stressor occurs at different times in the life-course. It will be interesting to investigate whether there are other genomic perturbations conserved across different dietary mouse models that may differ in their nature depending on the timing of the stress exposure [30]. Finally, it has been shown that tRNA fragments in sperm can cause gene expression changes in the livers of the offspring of sires exposed to PR during adulthood only [7, 8], which raises the question of whether inter-generational effects would be seen in the offspring of the PRPR group where no tRNA or other small RNA response is seen. The result supports the idea that small RNA in sperm are reflective of the paternal state [31], which in this case is the absence of an acute stress response due to long-term exposure to the stressor.

## Conclusions

To conclude, we show that the nature of the molecular response to PR is different depending on the timing of the exposure and that ‘matching’ diets either side of weaning eliminates responses measured at rDNA and small RNA. It is important to emphasise that both rDNA and small RNA stress responses are, broadly speaking, conserved amongst different species [15–20] and in different nutritional stress models [2, 8, 32, 33]. Our work supports the idea that genetic–epigenetic interactions at rDNA and small RNA could have utility as biomarkers to study key aspects of human biology and disease and how environmental pressures during the entire life-course could impact outcomes.

## Methods

### Breeding and housing conditions

All animal procedures were conducted in accordance with the Home Office Animals (Scientific Procedures) Act 1986 (Project License number: 70/6693). Female and male C57BL/6 J mice were obtained from Charles Rivers UK, aged 6–8 weeks and 10 weeks, respectively. Mice were maintained on a 12 h light/dark cycle (07:00–19:00) and housed at a constant temperature and humidity. After 1 week of acclimatisation in the mouse facility on standard chow (control diet, 20% protein), matings were set up by transferring one, or sometimes two, females into a male’s cage in the late afternoon. On the discovery of a vaginal plug the next morning (designated 0.5 days post coitum), pregnant females were singly housed and given ad libitum access to either a PR diet (8% protein) or maintained on the CT diet. Breeding males were housed individually for the duration of the breeding period. Females were maintained on the respective diet until offspring were weaned. Whole litters were weighed at 7 and 14 days. Upon weaning at 21 days, male offspring from each litter were put on either a CT or PR diet until death. Only litters with 5–10 pups were included. Litter sizes had no impact on the conclusions reported here (Additional file 1: Figures S8 and S9). Male offspring were housed in cages containing 3–5 mice from weaning and weighed individually every week from weaning until they were killed at 11–13 weeks of age.

### Diets

The CT diet was PicoLab^®^ Mouse Diet 20 Extruded (5R58*), consisting of a standard chow containing roughly 20% of calories from protein. The PR diet was a custom diet obtained from Special Diet Services and was isocaloric with the control diet but contained only 8% of calories from protein (code: 829277, name: RB 8% CP ISO E (P)) (Diet compositions outlined in Additional file 1: Table S1).

### Adult male dissection and phenotyping

Mice were fasted for 16 h before being killed by CO_2_ asphyxiation. After weighing the whole animal and measuring its length from nose to base of the tail, cardiac puncture was performed using a 23 G needle and 1 mL syringe. Between 100 and 500 μL of blood was collected. A drop of blood from the syringe (0.6 μL) was placed on a glucose measurement strip and blood glucose concentration was measured using a Bayer NEXT Contour Glucose meter. The remaining blood was decanted into a 1.5 mL Eppendorf tube and allowed to clot at room temperature before being placed on ice. In male mice, the epididymis was next dissected from the base of the testes and transferred to a 2 mL Eppendorf tube containing pre-made sperm motility medium warmed to 37 °C in a water bath (sperm motility medium: 1 M NaCl, 100 mM KCl, 25 mM KH_2_PO_4_, 20 mM MgSO_4_, 0.6% sodium lactate, 500 mM NaHCO_3_, 25 mM sodium pyruvate, 25 mM CaCl, 500 mM HEPES, 34.5 mg/mL of BSA). Epididymides were homogenised using a fine pair of scissors in the tube for 5 min and placed in a water bath for 30 min at 37 °C, with regular inversion, to allow the sperm to swim out. After incubation, the tube was briefly spun down using a nanofuge to collect debris, then the supernatant containing free-swimming sperm was removed and placed in a new 1.5 mL Eppendorf tube and stored on ice for the rest of the dissection.

Liver, kidneys and visceral gonadal white adipose tissue deposits were dissected out, weighed and flash frozen in liquid nitrogen. A small amount of pancreas and small intestine were also removed and flash frozen. Next, the subcutaneous inguinal white adipose tissue deposits on each side of the mouse and the interscapular brown adipose tissue deposits on the back of the mouse were removed, weighed and flash frozen. Finally, a small section of ear was flash frozen.

### Phenol:chloroform DNA extraction

For DNA extraction, one-quarter of the extracted sperm was incubated overnight in 600 μL of PK buffer (10 mM Tris-HCl, 100 mM NaCl, 25 mM EDTA, 1% SDS) with 2 μL of Proteinase K enzyme (19 mg/mL) and 0.1 M DTT at 55 °C with slow rotation. Phenol (750 μL) was added to the samples and agitated for several minutes before spinning at 17,000x *g* for 5 min at 4 °C. The upper aqueous phase was transferred to a new tube and the process repeated with phenol:chloroform, then chloroform alone. After the final spin, 5 μL of Rnase was added and samples were incubated at 37 °C for 60 min. Then, 0.1 volumes of 3 M sodium acetate (pH 5.2) and 2.5 volumes of 100% EtOH were added and samples incubated at −20 °C for 1 h. Samples were spun at 17,000x *g* for 10 min at 4 °C and the pellet was washed with 75% EtOH. Finally, the pellet was air-dried at 37 °C, resuspended in 200 μL of TE buffer and incubated at 50 °C for 3 h before storage at 4 °C. DNA was quantified using the High Sensitivity Qubit^®^ kit (Thermo Fish Scientific, Cat. Q32851) as per the protocol. Sperm purity was confirmed by Bis-PCR-Seq of imprinting control regions associated with *MEST*, *MCTS2*, *NESP* and *IGF2/H19*.

### RNA extraction and small RNA library preparation

For RNA extraction, three-quarters of the extracted sperm were incubated at 60 °C for 15 min with slow rotation in 33.3 μL of sperm lysis buffer (6.4 M Guanidine HCl, 5% Tween 20, 5% Triton, 120 mM EDTA, 120 mM Tris; pH 8.0) with 3.3 μL of Proteinase K (19 mg/mL) and 3.3 μL of 0.1 M DTT. After the incubation, one volume (100 μL) of ultra-pure water was added, followed by 700 μL of Qiazol Lysis reagent (QIAGEN, Cat. 79,306) and samples were vortexed for 5 min. Chloroform (140 μL) was added and samples were shaken vigorously for 30 s before 3 min incubation at room temperature. Samples were centrifuged at 12,000x *g* at 4 °C for 15 min then the upper aqueous phase was transferred to a new reaction tube. One volume of 70% EtOH was added and mixed thoroughly. Samples were transferred to a RNeasy mini spin column and the protocol from the miRNeasy Mini Kit (Qiagen, Cat. 217,004) was then followed, including the separation of the small RNA and large RNA fractions using an RNA MinElute spin column (Qiagen, Cat. 74,204). The small RNA fraction was eluted in 14 μL of RNase-free water and quantified using the microRNA kit from Qubit^®^ (Cat. Q32880). Them, 6 μL of the small RNA fraction was used for small RNA library preparation using the NEBNext^®^ Small RNA library prep set for Illumina (Cat. E7330S) as per the protocol. Each sample was uniquely barcoded using one of the NEBNext^®^ Index Primers for Illumina (Cat. E7300S, E7580S, E7710S, E7730S). For PCR amplification, 15 cycles were used. Libraries were purified using the QIAQuick PCR purification kit (Qiagen, Cat. 28,104) and DNA was eluted into 32 μL of nuclease-free water. An aliquot of each library was diluted and 1 μL was run on an Agilent 2100 Bioanalyzer using the Agilent High Sensitivity DNA kit (Cat. 5067-4626) to assess the size distribution of the library. The libraries were pooled using equal volumes and size selected for between 140 and 200 bp (corresponding to an insert size of 13–73 bp) using a BluePippin machine (Sage Science) with 3% agarose cassettes (Sage Science, Cat. BDF3010).

### Sequencing and data analysis

DNA from sperm was diluted to a concentration of 11 ng/μL and 45 μL of each sample was sent for sequencing to the Genome Centre Facility at Charterhouse Square, QMUL. Bis-PCR-Seq was performed using the 48.48 layout on the Fluidigm^®^ C1 system (Fluidigm^®^, USA), coupled with Illumina MiSeq sequencing using version2 chemistry (150 bp, paired-end). See Additional file 1: Table S3 for the primer sequences. Small RNA libraries were initially sequenced using Illumina MiSeq Nano sequencing (75 bp, single-end) and read counts for each samples were used to re-balance the library pool. The final pool was then sequenced using Illumina NextSeq sequencing (75 bp, single-end).

Bismark (v0.7.12) was used to align Bis-PCR-Seq data to the mm10 reference genome (imprinting control region data; Additional file 1: Figure S4) or to the adjusted consensus rDNA reference, using Bowtie2 (v2.1.0). Only reads that mapped to the correct starting position and perfectly matched the consensus were used for further analysis. For rDNA analysis, the R package RSamtools was used to identify each read as either having an A or a C at position −104 bp and to determine the methylation status at position −133 bp of each read. Reads could therefore be assigned to either A^m^, A^u^, C^m^, or C^u^. The total number of reads in each group was summed and %A ((A^m^ + A^u^)/(C^m^ + C^u^)) and CpG –133 A meth % (A^m^/(A^m^ + A^u^)) calculated for each sample. Methylation of imprinting control regions were assessed using a custom program (https://bitbucket.org/lowelabqmul/methylation-extractor).

Small RNA sequencing data was mapped to the whole genome (UCSC, mm10), piRNA, tRNA, miRNA and rRNA databases using the SPORTS 1.0 pipeline (https://github.com/junchaoshi/sports1.0.git). Total read counts for each small RNA class were expressed as percentages of number of reads mapped to the genome for each sample in the composition analysis (Fig. 4a). Differential expression analysis of tRNA fragments was performed using edgeR (glmQLFTest) using the number of reads mapping to the genome as the library sizes for normalisation.

### Statistics

All statistical analysis and plotting were performed using R (v3.2.3). For all phenotype plots, a linear model was run on individuals and *P* values were derived by using robust standard errors to account for the relatedness between siblings in each diet group (R packages *plm* [34] and *lmtest* [35]) and corrected for *n* = 3 tests using Bonferroni’s correction. A Pearson’s product moment correlation coefficient was calculated to describe the relationship between %A and CpG –133 A methylation percentage in each diet group (cor) and a linear model with robust standard errors was then run to obtain *P* values, which were then corrected for *n* = 4 comparisons using Bonferroni’s correction (*P*_*lin*_). All mice were used in the analyses instead of litter averages and linear models with robust standard errors were used to correct for any biases due to sibling relatedness (a full justification is provided in Additional file 1: Supplementary methods). Fisher’s exact test was used to assess the differences in distribution of the small RNA compositions compared to CTCT, using percentages of mapped reads corresponding to each species in each sample and these were rounded to the nearest integer (corrected for *n* = 3 tests using Bonferroni’s correction). ANOVA was used to assess whether the %A or %C and CpG –133 A meth % or CpG –133 C meth % were different between the diet groups.

## Additional file

Additional file 1: **Table S1.** Compositions of the control (CT) diet and the protein restricted (PR) diet. **Table S2.** List of all male mice studied with each litter represented by the first two numbers and letter of each ID. **Table S3.** Sequences of primers used for targeted analysis of DNA methylation at rDNA CpG −133 and the imprinted regions *MEST*, *MCTS2*, *NESP* and *IGF2/H19*. **Figure S1.** Growth rates of mice and lengths at death in each group. **Figure S2.** Absolute and relative organ weights. **Figure S3.** Absolute and relative adipose tissue deposit weights. **Figure S4.** Sperm small RNA size distribution analysis and sperm purity analysis. **Figure S5.** Read length distribution of reads mapping to the genome that also map to different classes of small RNA, normalised by total number of reads mapping to the genome. **Figure S6.** Maternal weight, food intake and litter size data. **Figure S7.** %A/C and CpG –133 meth % distribution in four diet groups. **Figure S8.** Growth trajectories plotted by pre-weaning litter size (including females). **Figure S9.** Pre-weaning litter size (including females) has no impact on CpG –133 A meth % in any of the groups. **Supplementary methods.** Rationale and explanation of the use of a linear model and robust standard errors to analyse the relationship between %A and CpG –133 A meth % instead of using litter averages or individuals from the same litter without correction for relatedness. R script used to perform the analysis is also included. (DOCX 1338 kb)
