# Supplementary material for: Early life diet conditions the molecular response to post-weaning protein restriction in the mouse
Source: BMC Biol. 2018 May 2;16:51. doi: 10.1186/s12915-018-0516-5 (PMC5930764; doi:10.1186/s12915-018-0516-5)
Supplement: Supplementary file 1 — Table S1. Compositions of the control (CT) diet and the protein restricted (PR) diet. Table S2. List of all male mice studied with each litter represented by the first two numbers and letter of each ID. Table S3. Sequences of primers used for targeted analysis of DNA methylation at rDNA CpG −133 and the imprinted regions MEST, MCTS2, NESP and IGF2/H19. Figure S1. Growth rates of mice and lengths at death in each group. Figure S2. Absolute and relative organ weights. Figure S3. Absolute and relative adipose tissue deposit weights. Figure S4. Sperm small RNA size distribution analysis and sperm purity analysis. Figure S5. Read length distribution of reads mapping to the genome that also map to different classes of small RNA, normalised by total number of reads mapping to the genome. Figure S6. Maternal weight, food intake and litter size data. Figure S7. %A/C and CpG –133 meth % distribution in four diet groups. Figure S8. Growth trajectories plotted by pre-weaning litter size (including females). Figure S9. Pre-weaning litter size (including females) has no impact on CpG –133 A meth % in any of the groups. Supplementary methods. Rationale and explanation of the use of a linear model and robust standard errors to analyse the relationship between %A and CpG –133 A meth % instead of using litter averages or individuals from the same litter without correction for relatedness. R script used to perform the analysis is also included. (DOCX 1338 kb) [file 12915_2018_516_MOESM1_ESM.docx]

Additional file

| **Component (10% H2O)** | **CT diet** | **PR diet** |
| --- | --- | --- |
| Protein % | 21.9 | 7.48 |
| Arg | 1.14 | 0.23 |
| Cys | 0.31 | 0.14 |
| Gly | 0.93 | 0.29 |
| His | 0.5 | 0.17 |
| Iso | 0.99 | 0.37 |
| Leu | 1.81 | 0.55 |
| Lys | 1.11 | 0.46 |
| Met | 0.67 | 0.18 |
| Phe | 0.96 | 0.32 |
| Tyr | 0.63 | 0.3 |
| Thr | 0.78 | 0.25 |
| Trp | 0.25 | 0.07 |
| Val | 1.02 | 0.44 |
| Ser | 1.04 | 0.27 |
| Asp | 2.07 | 0.41 |
| Glu | 4.39 | 1.18 |
| Ala | 1.31 | 0.23 |
| Pro | 1.52 | 0.5 |
| Taurine | 0.04 | 0 |
| Gross energy MJ/kg | 19.35 | 15.68 |
| Metabolisable energy MJ/kg | 14.95 | 13.13 |
| Crude fat % | 21% | 7.36 |
| Crude fibre % | 2.2 | 7.36 |
| Nitrogen free extract % | 51.5 | 67.07 |
| Starch % | 36.4 | 41.22 |
| Cellulose, lignin % | 3 | 4.51 |
| Hemicellulose % | 7.9 | 0.09 |
| Sugar % | 1.77 | 24.52 |
| Sucrose % | 0.67 | 24.52 |
| Ash % | 5 | 3.36 |
| Vitamins |  |  |
| Vit A iu/kg | 17000 | 4000 |
| Vit D3 iu/kg | 3600 | 1441.28 |
| Vit E iu/kg | 67 | 76.77 |
| Vit B1 mg/kg | 16 | 4.6 |
| Vit B2 mg/kg | 9.7 | 4.65 |
| Vit B6 mg/kg | 12 | 5.39 |
| Vit B12 ug/kg | 61 | 23.49 |
| Vit C | 0 | 0 |
| Vit K mg/kg or ppm | 3.1 | 0.72 |
| Folic Acid mg/kg | 3.3 | 1.83 |
| Choline mg/kg | 2640 | 1261.63 |
| Biotin ug/kg | 300 | 187.91 |

Table S1
Compositions of the control (CT) diet and the protein restricted (PR) diet.

| **ID** | **Diet** | **sperm DNA read count** | **%A** | **CpG-133 A meth %** | **small RNA library size** | **Number of small RNA reads mapped to genome** |
| --- | --- | --- | --- | --- | --- | --- |
| 07C3 | CTCT | 140398 | 42.77279 | 49.861955 | 11524746 | 6456120 |
| 07C4 | CTCT | 58772 | 40.78870497 | 40.6779661 |  |  |
| 07C5 | CTCT | 101481 | 41.28843338 | 53.06015235 |  |  |
| 07C6 | CTPR | 103728 | 43.8668 | 36.01859 |  |  |
| 02A6 | CTCT | 174647 | 45.20304902 | 13.88283181 | 10709017 | 4465281 |
| 02A7 | CTCT | 118006 | 42.67087451 | 22.55741534 |  |  |
| 02A8 | CTPR | 119659 | 45.70621 | 15.327565 | 10959561 | 4596770 |
| 09H5 | CTPR | 116531 | 47.29345 | 23.795181 | 11528930 | 6323869 |
| 09H6 | CTPR | 443022 | 42.2553816 | 44.33831191 |  |  |
| 09H7 | CTCT | 110509 | 47.62611 | 31.838006 | 11360218 | 6393098 |
| 35F1 | CTPR | 103071 | 49.63863 | 25.545996 | 12607711 | 6845493 |
| 35F2 | CTCT | 116211 | 45.62118 | 18.877551 | 11315092 | 6706672 |
| 29L4 | CTPR | 85402 | 43.4693 | 17.440758 |  |  |
| 29L5 | CTPR | 250379 | 46.81054558 | 5.863018566 |  |  |
| 29L6 | CTCT | 79205 | 42.31499 | 28.251121 | 10859504 | 6114067 |
| 45L1 | CTPR | 111284 | 46.53641 | 36.641221 | 12473826 | 7316533 |
| 45L2 | CTPR | 272614 | 46.82502352 | 24.04989118 |  |  |
| 45L3 | CTCT | 84531 | 48.22196 | 32.982172 | 11375392 | 6065268 |
| 45L4 | CTCT | 37980 | 42.75793651 | 17.46768313 |  |  |
| 53M1 | CTCT | 114807 | 50.22999 | 22.893773 | 11802003 | 6687238 |
| 53M2 | CTCT | 27439 | 48.87775551 | 41.04141041 |  |  |
| 53M3 | CTCT | 30458 | 45.06486182 | 27.40926158 |  |  |
| 53M4 | CTPR | 94669 | 48.44541 | 23.059701 | 11800058 | 6934403 |
| 53M5 | CTPR | 39071 | 47.17083787 | 23.67358708 |  |  |
| 56I1 | CTPR | 119132 | 46.51661 | 20.852535 | 12628920 | 6821362 |
| 47I1 | CTPR | 104749 | 44.71752 | 37.044968 | 11557791 | 6212725 |
| 47I2 | CTCT | 113857 | 44.38836 | 21.070234 | 11018817 | 6013156 |
| 49K1 | CTCT | 99451 | 46.91877 | 22.164179 | 10816169 | 6138080 |
| 49K2 | CTCT | 208459 | 43.5666797 | 29.17414722 |  |  |
| 49K3 | CTPR | 88896 | 48.00644 | 17.533557 | 12406813 | 6454719 |
| 57P1 | CTPR | 84851 | 42.73404 | 42.529789 | 12137972 | 6825363 |
| 57P2 | CTPR | 414830 | 43.51132686 | 42.67385645 |  |  |
| 57P3 | CTCT | 97822 | 44.96796 | 35.624476 | 11009463 | 6190762 |
| 14A4 | PRCT | 93090 | 43.53499 | 25.703906 | 11025512 | 5744388 |
| 14A5 | PRPR | 93178 | 43.39903 | 17.39503 | 12020947 | 5551605 |
| 08G3 | PRCT | 95458 | 41.46434 | 22.872827 | 11792098 | 6545391 |
| 08G4 | PRCT | 409312 | 42.49239635 | 10.56068312 |  |  |
| 08G5 | PRPR | 104839 | 57.22362 | 24.753019 | 12017752 | 6192989 |
| 08G6 | PRPR | 540543 | 52.9587031 | 23.76285541 |  |  |
| 08G7 | PRPR | 236575 | 44.69669938 | 18.96018361 |  |  |
| 23F4 | PRCT | 101213 | 50.785 | 33.817035 | 11375478 | 6573944 |
| 23F5 | PRCT | 108037 | 44.8397634 | 53.00255956 |  |  |
| 23F6 | PRPR | 87864 | 47.93037 | 35.835351 | 11535051 | 6711162 |
| 23F7 | PRPR | 62038 | 46.23900673 | 50.70485567 |  |  |
| 40H1 | PRCT | 84413 | 47.24959 | 48.566464 | 12758010 | 6991107 |
| 40H2 | PRCT | 33170 | 43.69031378 | 32.31850117 |  |  |
| 40H3 | PRPR | 103309 | 47.52343 | 35.774648 | 11839308 | 6361569 |
| 31A3 | PRCT | 57483 | 43.1435 | 11.01 | 11340635 | 6031923 |
| 31A4 | PRCT | 59645 | 38.27246492 | 14.58658346 |  |  |
| 31A5 | PRCT | 55889 | 43.09162451 | 29.27211062 |  |  |
| 31A6 | PRPR | 84255 | 49.64716 | 38.210702 | 12514514 | 6858901 |
| 39F5 | PRCT | 84561 | 44.91726 | 49.912281 | 11912007 | 6519785 |
| 39F6 | PRPR | 105420 | 46.62056 | 46.392448 | 12167128 | 6286353 |
| 36K3 | PRPR | 112522 | 45.75284 | 45.77748 | 11793513 | 6104685 |
| 46I1 | PRCT | 102295 | 43.14231 | 23.731343 | 11553319 | 6473827 |
| 46I2 | PRCT | 296496 | 38.09802121 | 18.56234933 |  |  |
| 46I3 | PRPR | 84671 | 42.73469 | 27.316141 | 11703292 | 5815195 |
| 46I4 | PRPR | 480888 | 39.93704937 | 27.00696481 |  |  |
| 51O1 | PRCT | 94665 | 38.43762 | 24.064712 | 11015874 | 5894814 |
| 51O2 | PRCT | 76762 | 39.38680878 | 31.06659638 |  |  |
| 51O3 | PRPR | 108654 | 39.80084 | 51.402578 | 10834482 | 5973305 |
| 59L1 | PRCT | 130186 | 47.06584 | 25.646224 | 13496750 | 7612603 |
| 59L2 | PRCT | 53711 | 44.10232117 | 41.84747583 |  |  |
| 59L3 | PRPR | 97822 | 41.14187 | 29.857023 | 10898212 | 6175850 |

Table S2
List of all male mice studied with each litter represented by the first two numbers and letter of each ID. Diet groups and read counts for bisulfite sequencing and small RNA-seq are indicated as well as the number of small RNA reads that matched to the genome, which was used for normalisation and further analyses. Individuals from each diet group from each litter used for small RNA analysis are indicated. Raw data for %A and CpG-133 A meth % are shown.

| **Target** | **RefSeq ID** | **FP (5'-3')** | **RP (5'-3')** |
| --- | --- | --- | --- |
| CpG-133 | BK000964 | tgttaggtYGattagttgttttt | tatcaatacctatctccaaatcca |
| MEST | NM_008590 | TTTGGATTTATAATGGTAGGGTTA | ATTTATCTAATACCACCAAACCAA |
| MCTS2 | NM_025543 | AGTATTAGAATATTGGGGGATTTT | TACCCCACTAATTCTTCTTCAAAT |
| NESP | AF175305 | ATAGTGGATAGTGTTTGGAGGATA | CCAAAACCACTTCTTATTACTCTC |
| IGF2/H19 | NM_001122737 | ATGGGATTATAGATGGTGATAGG | AAACAAAACACATACATTTTCTAAAC |

Table S3
Sequences of primers used for targeted analysis of DNA methylation at rDNA CpG -133 and the imprinted regions MEST, MCTS2, NESP and IGF2/H19.


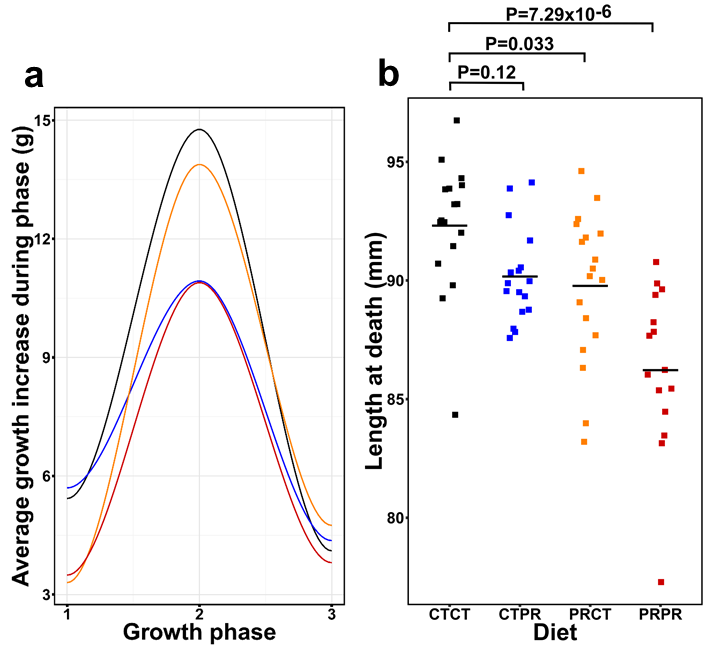


Figure S1
**a** Mean growth increase in each diet group during growth phase 1 (7-21 days of age), growth phase 2 (21-42 days of age) and growth phase 3 (42-63 days of age). After weaning at 21 days, growth increase is the same in post-weaning diet groups. **b** Lengths at death were significantly different from controls in the PRCT and PRPR groups.


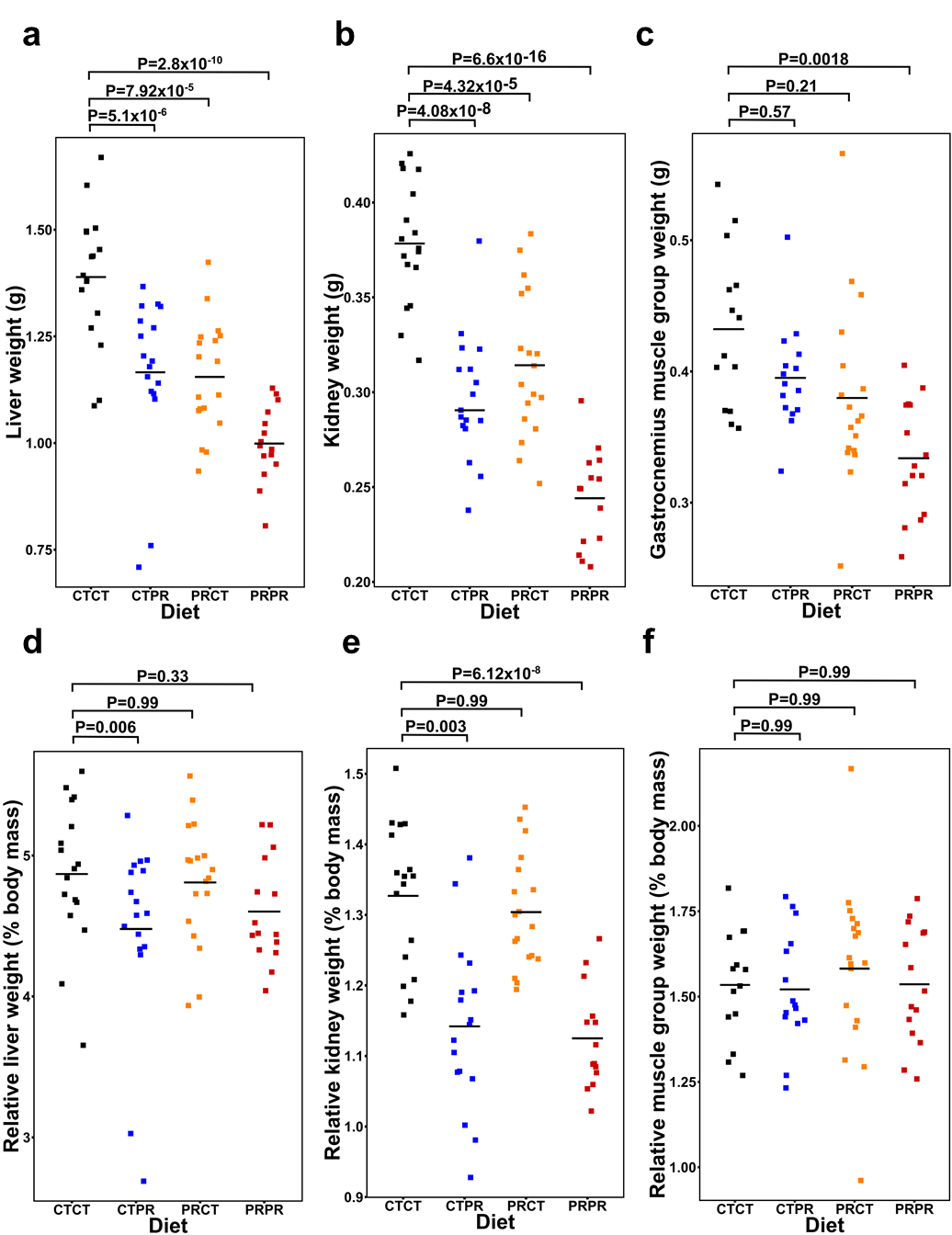


Figure S2 **a** Absolute liver weights were lower in the CTPR, PRCT and PRPR groups compared with the CTCT group. **b** Absolute kidney weights (weight of both kidneys combined) were lower in the CTPR, PRCT and PRPR groups than in the CTCT group, although CTPR kidney weights were lower than PRCT weights. **c** Gastrocnemius muscle group absolute weight tracked with the body weights at death. **d** Relative liver weights were lower in the CTPR group but the same as controls in PRCT and PRPR. **e** Relative kidney weights (both kidneys combined) were the same between CTCT and PRCT but CTPR and PRPR groups had lower relative kidney weights. **f** The relative muscle weights were the same between the four diet groups.


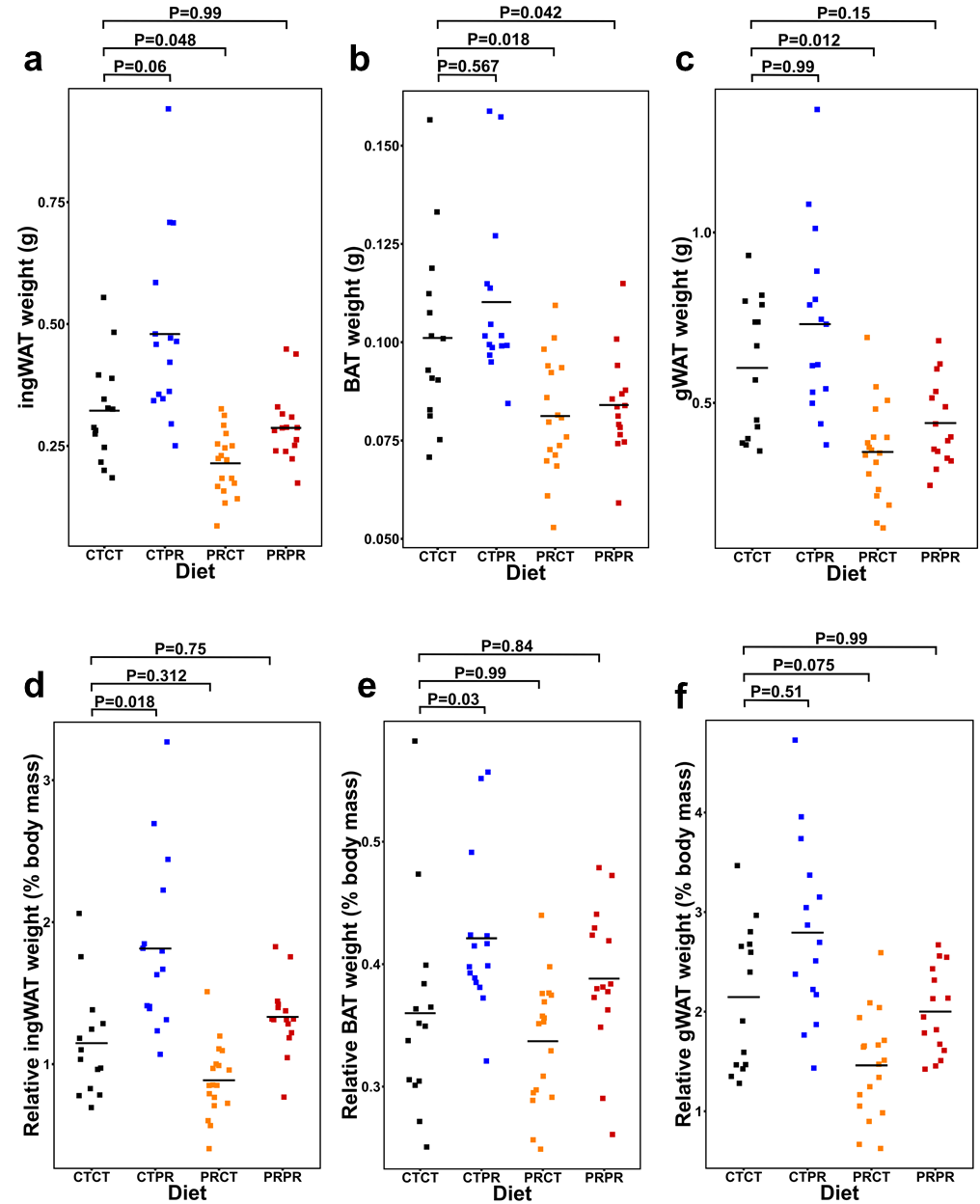


Figure S3 **a** Absolute weight of the two subcutaneous inguinal white adipose tissue deposits (ingWAT) was lower in PRCT but the same in CTPR and PRPR as the CTCT group. **b** Absolute weight of the interscapular brown adipose tissue deposit (BAT) was the same in CTPR and lower in PRCT and PRPR than in the CTCT group. **c** Absolute weight of the perigonadal visceral white adipose tissue deposit (gWAT) was the same in CTPR and PRPR, and lower in PRCT than CTCT group. **d** ingWAT weight relative to body mass was higher in CTPR, but the same in PRCT and PRPR compared to the CTCT group. **e** BAT weight relative to body mass was higher in CTPR and the same in PRCT and PRPR as the CTCT group. **f** gWAT weight relative to body mass was the same between groups.


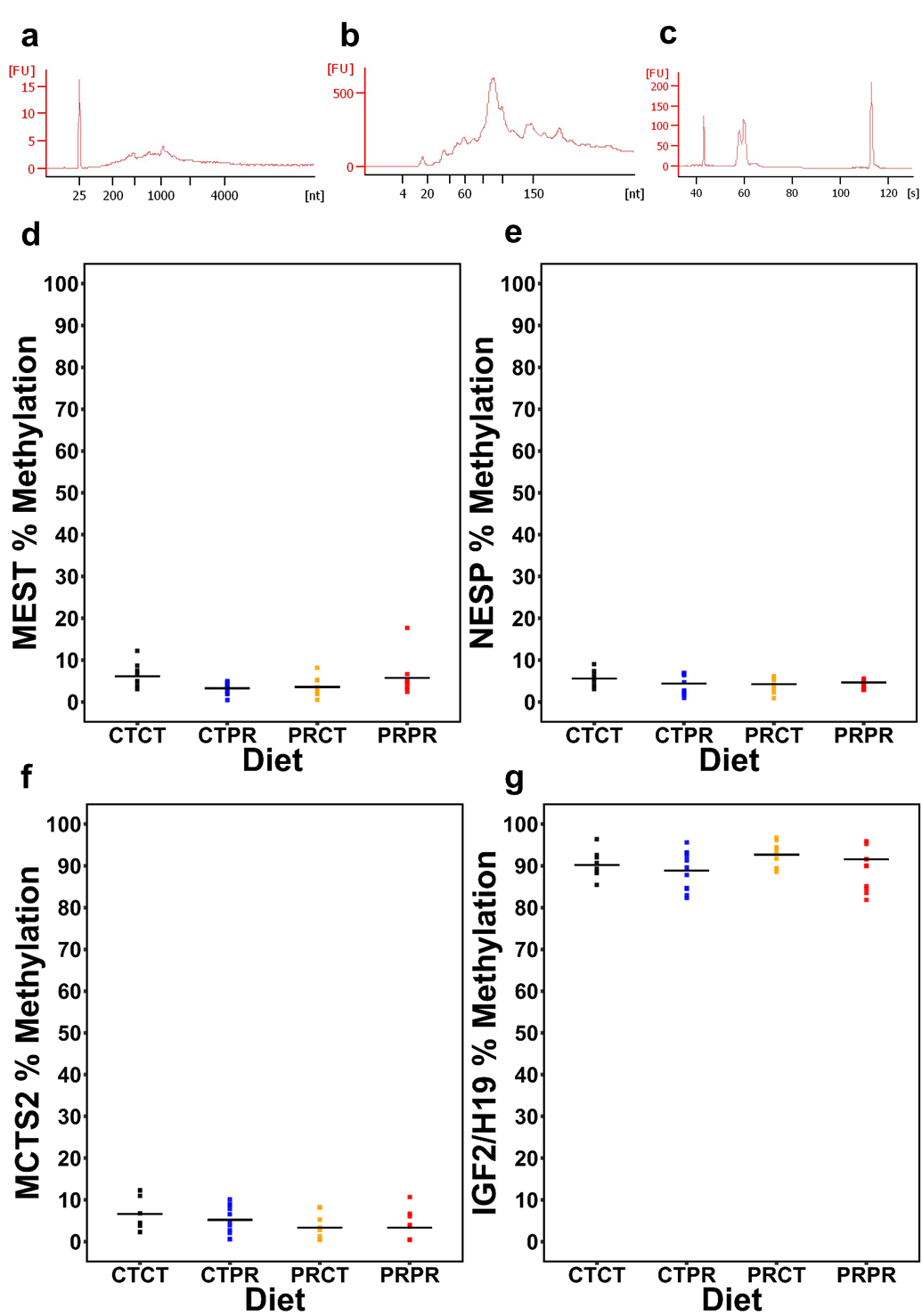
Figure S4
**a** Representative sperm small RNA trace from Agilent Bioanalyser RNA 6000 Nano chip, showing a lack of somatic cell contamination due to the absence of ribosomal RNA peaks. **b** Representative sperm small RNA trace from Agilent Bioanalyser Small RNA chip. **c** Representative small RNA library trace from Agilent Bioanalyser High Sensitivity DNA chip after size selection. **d** High sperm purity is demonstrated in all four groups by low methylation levels at the three imprinting control regions MEST,(**e**) NESP and (**f**) MCTS2 but high methylation levels at the imprinting control region IGF2/H19 (**g**).


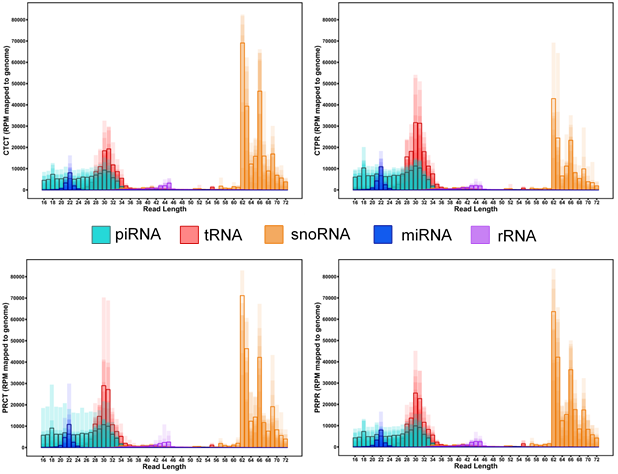


Figure S5
Read length distribution of reads mapping to the genome that also map to different classes of small RNA, normalised by total number of reads mapping to the genome. Each sample is plotted and overlaid. Group averages are represented by the darker coloured lines.


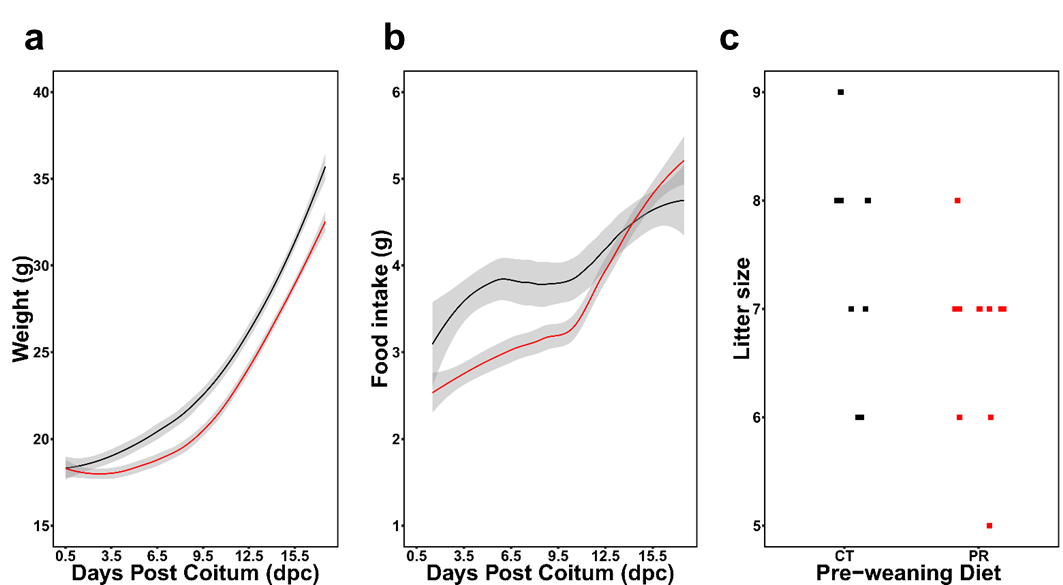


Figure S6
**a** Weight progression of pregnant females fed either a CT diet (black, n=8) or a PR diet (red, n=9) between 0.5 and 17.5 dpc. Coloured line = loess curve; span=0.6, grey area = 95% confidence limits. **b** Food intake of pregnant females fed either a CT diet (n=10) or PR diet (n=13) measured between 1.5 and 17.5 dpc. Coloured line = loess curve; span=0.7, grey area = 95% confidence limit. **c** Litter size is the same between CT and PR exposed mothers (Welch’s t-test, P=0.079).


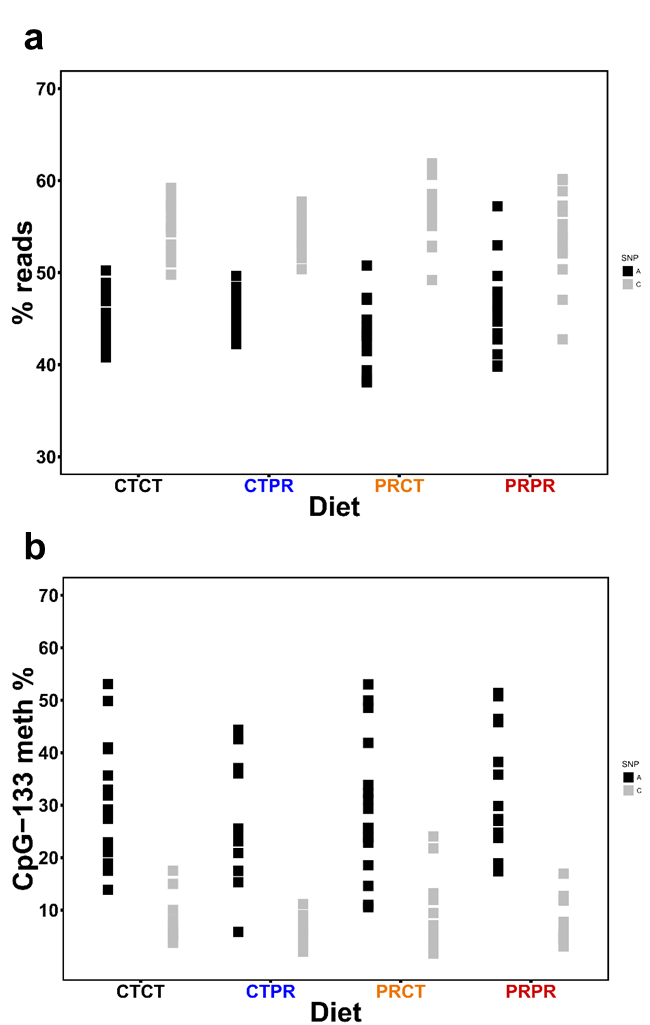
Figure S7
**a** Percentage of reads with an ‘A’ at position -104 (black) and ‘C’ at position -104 (grey) in the ribosomal DNA in each diet group. %A is the reciprocal of %C. %A and %C are the same between the groups (ANOVA; P=0.06 and P=0.06 respectively). **b** Percentage of ‘A reads’ that are methylated at position -133 (black) compared with the percentage of ‘C reads’ that are methylated at position -133 (grey) in each diet group. CpG-133 A meth % and CpG-133 C meth % are the same between the groups (ANOVA; P=0.5 and P=0.39 respectively).

Figure S8
Growth trajectories plotted by pre-weaning litter size (including females). Points represent individual weight measurements of each mouse. Grey shaded area represents the 95% confidence boundary for the data represented by the coloured line. Although there is variation in growth rate depending on the size of the litter, t
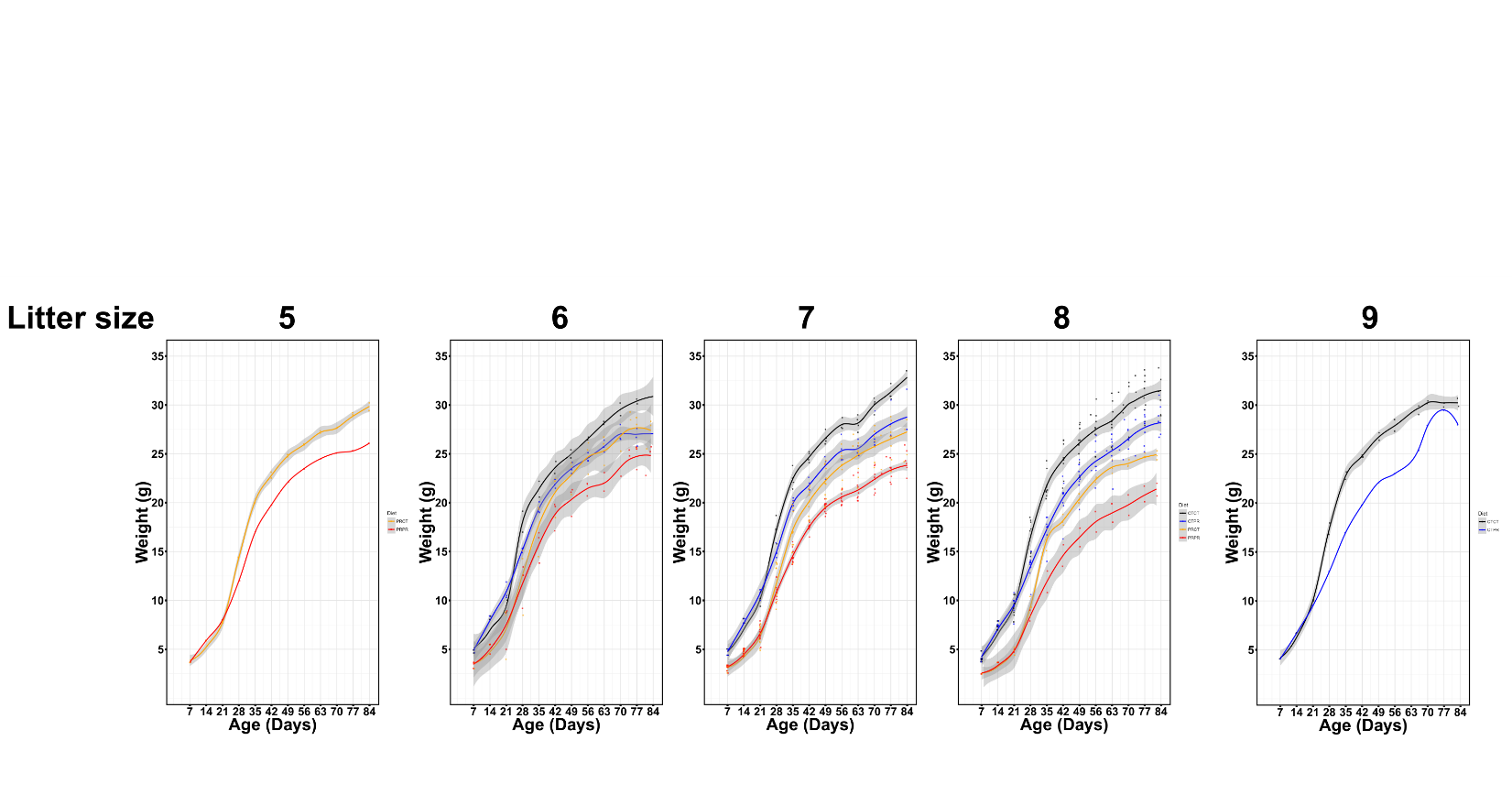
he growth trajectories of the four groups remain distinct.


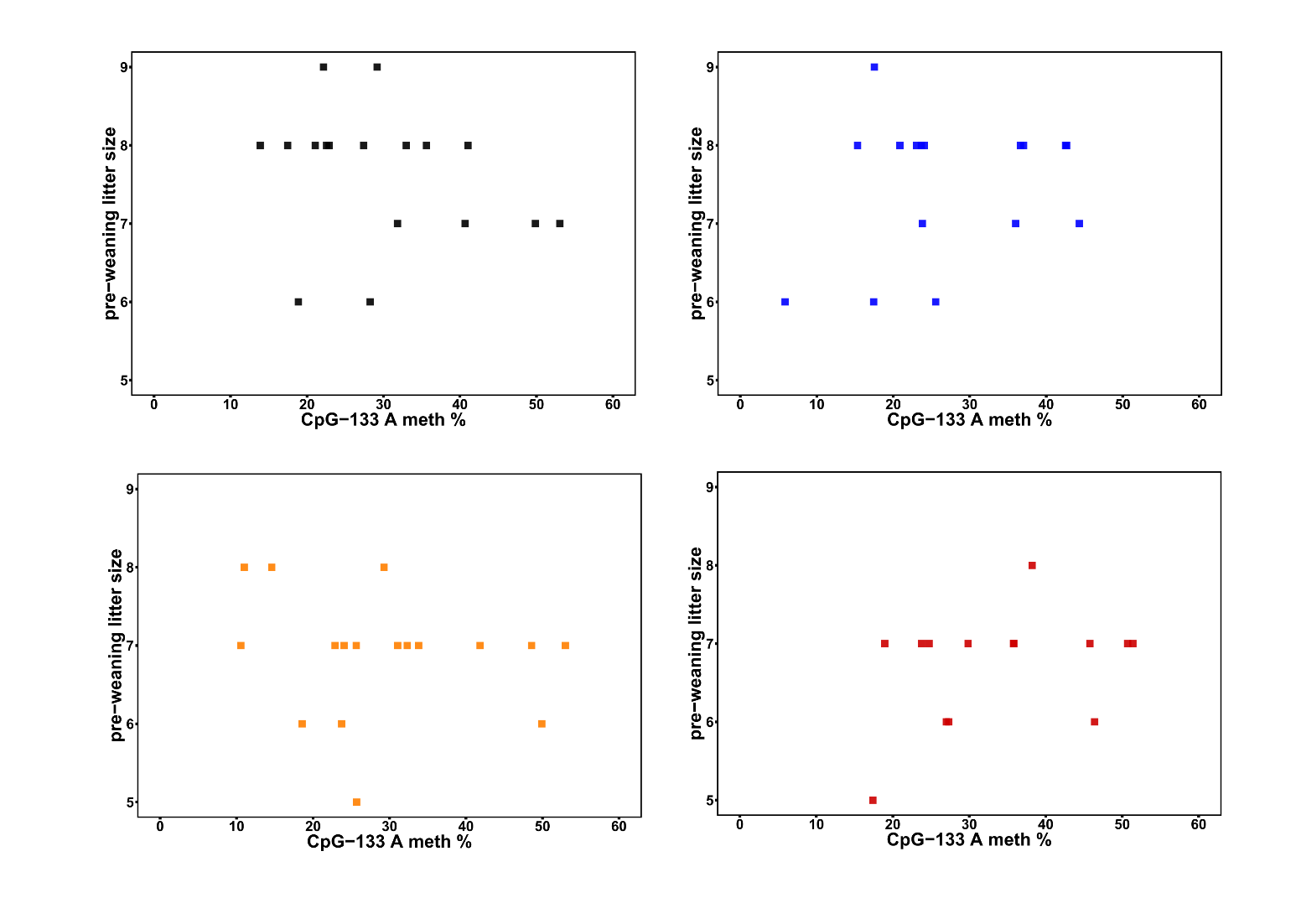


Figure S9
Pre-weaning litter size (including females) has no impact on CpG-133 A meth % in any of the groups. Despite variation in litter sizes, there is no difference overall between the litter sizes in the pre-weaning CT and PR group and litter size is not a confounding factor in determining CpG-133 A meth %.

Supplementary methods


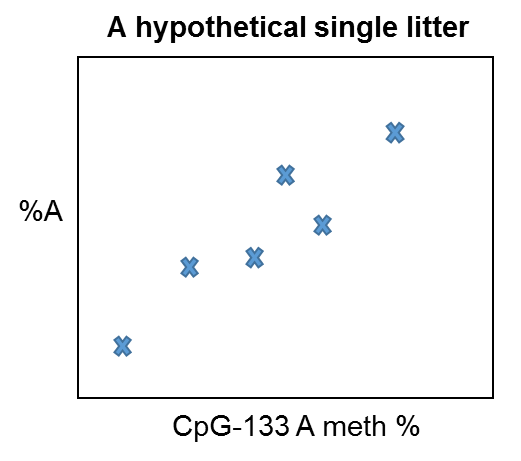
For figure 3, we have included all of the mice that were used in the experiment, some of which were siblings that were on the same dietary regimes, to assess the relationship between %A and CpG-133 A meth %. When multiple siblings are used in an analysis such as this, it is commonplace to take a litter average. Had we performed Pearson’s product moment correlation tests on the litter averages of %A and CpG-133 A meth % in the four groups, the results would have been CTCT cor=-0.267, P=0.4567 (P_adjust_=0.99); CTPR cor=-0.516, P=0.1038 (P_adjust_=0.4152); PRCT cor=0.73, P=0.02549 (P_adjust_=0.101), PRPR cor=-0.086, P=0.8139 (P_adjust_=0.99). However, the hypothetical plot below illustrates why this would not be appropriate in this case. %A and CpG-133 A meth % vary between siblings (despite these mice being an inbred strain) and taking an average would have meant the possibility of losing a relationship such as the one in the hypothetical plot below within a litter, and reducing the amount of usable data.

On the other hand, if we had treated all of the points as if they were independent, as would have been the case if we had run a Pearson’s product moment correlation test or a simple linear regression on all individuals, there could have been biases in the data caused by clustering of siblings, that could have resulted in an incorrect evaluation of the relationship between %A and CpG-133 A meth %. To correct for this, we used a linear model with robust standard errors to calculate the P values. This takes into account the clusters of siblings and corrects for them. When using this method, P values can become less significant (as in the CTPR group) or more significant (as in the PRCT group) depending on the data. The R script used to perform this analysis is outlined below.

We use the Pearson’s product moment correlation coefficient as a purely descriptive value because correlation coefficients are more easily interpreted than effect sizes. The P value does not therefore relate to the correlation coefficient, but to the linear model with robust standard errors analysis (signified by Plin).

R script for linear model with robust standard errors and calculation of correlation coefficient.

#libraries for robust standard error calculation

library**(**plm**)**

library**(**lmtest**)**

#data read into R

data**<-**read.csv**(**"/path/to/data/supplementary/table/"**)**

#data subsetted by diet group

PRCT**=**subset**(**data, diet**==**"PRCT"**)**

CTCT**=**subset**(**data, diet**==**"CTCT"**)**

PRPR**=**subset**(**data, diet**==**"PRPR"**)**

CTPR**=**subset**(**data, diet**==**"CTPR"**)**

#Pearson's product moment correlation calculated to describe the relationship between %A and CpG-133 A meth%

cor.test**(**CTCT**$**sperm_cpg133a, CTCT**$**sperm_aperc**)**

# Pearson's product-moment correlation

#data: CTCT$sperm_cpg133a and CTCT$sperm_aperc

#t = -1.0081, df = 15, p-value = 0.3294

#alternative hypothesis: true correlation is not equal to 0

#95 percent confidence interval:

# -0.6534320 0.2602533

#sample estimates:

# cor

#-0.2519017

cor.test**(**CTPR**$**sperm_cpg133a, CTPR**$**sperm_aperc**)**

#Pearson's product-moment correlation

#data: CTPR$sperm_cpg133a and CTPR$sperm_aperc

#t = -2.7366, df = 14, p-value = 0.01606

#alternative hypothesis: true correlation is not equal to 0

#95 percent confidence interval:

# -0.8401796 -0.1337847

#sample estimates:

# cor

#-0.59034

cor.test**(**PRCT**$**sperm_cpg133a, PRCT**$**sperm_aperc**)**

#Pearson's product-moment correlation

#data: PRCT$sperm_cpg133a and PRCT$sperm_aperc

#t = 2.2426, df = 15, p-value = 0.04046

#alternative hypothesis: true correlation is not equal to 0

#95 percent confidence interval:

# 0.0269279 0.7911802

#sample estimates:

# cor

#0.5010872

cor.test**(**PRPR**$**sperm_cpg133a, PRPR**$**sperm_aperc**)**

#Pearson's product-moment correlation

#data: PRPR$sperm_cpg133a and PRPR$sperm_aperc

#t = -0.5049, df = 12, p-value = 0.6228

#alternative hypothesis: true correlation is not equal to 0

#95 percent confidence interval:

# -0.6268383 0.4183677

#sample estimates:

# cor

#-0.1442269

####calculation of P values using linear model with robust standard errors####

#This is done because the Pearson's product moment correlation coefficent does not account for relatedness between siblings in the same diet group which could cause biases in the data.

###CTCT###

# Calculate a normal linear model first

model **<-** lm**(**CTCT**$**sperm_aperc **~** CTCT**$**sperm_cpg133a**)**

summary**(**model**)$**coefficients**[**"CTCT$sperm_cpg133a",**]**

# Calculate the clustered robust variance estimator

dat**<-**data.frame**(**"DNA.meth" **=** CTCT**$**sperm_cpg133a, "Percent.A" **=** CTCT**$**sperm_aperc, "litter" **=** CTCT**$**dam, "ID" **=** CTCT**$**ID**)**

p.dat**<-**pdata.frame**(**dat, index **=** c**(**"litter", "ID"**)**, drop.index **=** F, row.names **=** T**)**

pm1 **<-** plm**(**Percent.A **~** DNA.meth, model **=** "pooling", data **=** p.dat, na.action **=** na.omit **)**

G **<-** length**(**unique**(**CTCT**$**dam**))**

N **<-** length**(**CTCT**$**dam**)**

dfa **<-** **(**G**/(**G **-** 1**))** ***** **(**N **-** 1**)/**pm1**$**df.residual

firm_c_vcov **<-** dfa ***** vcovHC**(**pm1, type **=** "HC0", cluster **=** "group", adjust **=** T**)**

coeftest**(**pm1, vcov **=** firm_c_vcov**)[**"DNA.meth", c**(**1,2,4**)]**

# Estimate Std. Error Pr(>|t|)

#-0.06168394 0.06615916 0.36591745

#Bonferroni's correction for n=4 tests P=0.99

###CTPR###

# Calculate a normal linear model first

model **<-** lm**(**CTPR**$**sperm_aperc **~** CTPR**$**sperm_cpg133a**)**

summary**(**model**)$**coefficients**[**"CTPR$sperm_cpg133a",**]**

# Calculate the clustered robust variance estimator

dat**<-**data.frame**(**"DNA.meth" **=** CTPR**$**sperm_cpg133a, "Percent.A" **=** CTPR**$**sperm_aperc, "litter" **=** CTPR**$**dam, "ID" **=** CTPR**$**ID**)**

p.dat**<-**pdata.frame**(**dat, index **=** c**(**"litter", "ID"**)**, drop.index **=** F, row.names **=** T**)**

pm1 **<-** plm**(**Percent.A **~** DNA.meth, model **=** "pooling", data **=** p.dat, na.action **=** na.omit **)**

G **<-** length**(**unique**(**CTPR**$**dam**))**

N **<-** length**(**CTPR**$**dam**)**

dfa **<-** **(**G**/(**G **-** 1**))** ***** **(**N **-** 1**)/**pm1**$**df.residual

firm_c_vcov **<-** dfa ***** vcovHC**(**pm1, type **=** "HC0", cluster **=** "group", adjust **=** T**)**

coeftest**(**pm1, vcov **=** firm_c_vcov**)[**"DNA.meth", c**(**1,2,4**)]**

# Estimate Std. Error Pr(>|t|)

#-0.11407980 0.04958822 0.03731190

#Bonferroni's correction for n=4 tests P=0.15

####PRCT####

# Calculate a normal linear model first

model **<-** lm**(**PRCT**$**sperm_aperc **~** PRCT**$**sperm_cpg133a**)**

summary**(**model**)$**coefficients**[**"PRCT$sperm_cpg133a",**]**

# Calculate the clustered robust variance estimator

dat**<-**data.frame**(**"DNA.meth" **=** PRCT**$**sperm_cpg133a, "Percent.A" **=** PRCT**$**sperm_aperc, "litter" **=** PRCT**$**dam, "ID" **=** PRCT**$**ID**)**

p.dat**<-**pdata.frame**(**dat, index **=** c**(**"litter", "ID"**)**, drop.index **=** F, row.names **=** T**)**

pm1 **<-** plm**(**Percent.A **~** DNA.meth, model **=** "pooling", data **=** p.dat, na.action **=** na.omit **)**

G **<-** length**(**unique**(**PRCT**$**dam**))**

N **<-** length**(**PRCT**$**dam**)**

dfa **<-** **(**G**/(**G **-** 1**))** ***** **(**N **-** 1**)/**pm1**$**df.residual

firm_c_vcov **<-** dfa ***** vcovHC**(**pm1, type **=** "HC0", cluster **=** "group", adjust **=** T**)**

coeftest**(**pm1, vcov **=** firm_c_vcov**)[**"DNA.meth", c**(**1,2,4**)]**

#Estimate Std. Error Pr(>|t|)

#0.1319849465 0.0269692494 0.0001946462

#Bonferroni's correction for n=4 tests P=0.0008

####PRPR####

# Calculate a normal linear model first

model **<-** lm**(**PRPR**$**sperm_aperc **~** PRPR**$**sperm_cpg133a**)**

summary**(**model**)$**coefficients**[**"PRPR$sperm_cpg133a",**]**

# Calculate the clustered robust variance estimator

dat**<-**data.frame**(**"DNA.meth" **=** PRPR**$**sperm_cpg133a, "Percent.A" **=** PRPR**$**sperm_aperc, "litter" **=** PRPR**$**dam, "ID" **=** PRPR**$**ID**)**

p.dat**<-**pdata.frame**(**dat, index **=** c**(**"litter", "ID"**)**, drop.index **=** F, row.names **=** T**)**

pm1 **<-** plm**(**Percent.A **~** DNA.meth, model **=** "pooling", data **=** p.dat, na.action **=** na.omit **)**

G **<-** length**(**unique**(**PRPR**$**dam**))**

N **<-** length**(**PRPR**$**dam**)**

dfa **<-** **(**G**/(**G **-** 1**))** ***** **(**N **-** 1**)/**pm1**$**df.residual

firm_c_vcov **<-** dfa ***** vcovHC**(**pm1, type **=** "HC0", cluster **=** "group", adjust **=** T**)**

coeftest**(**pm1, vcov **=** firm_c_vcov**)[**"DNA.meth", c**(**1,2,4**)]**

# Estimate Std. Error Pr(>|t|)

#-0.06177019 0.12298078 0.62456351

#Bonferroni's correction for n=4 tests P=0.99
